# Skilled Nursing Facility Network Capacity and Hospital Length of Stay

**DOI:** 10.1001/jamanetworkopen.2026.9930

**Published:** 2026-04-30

**Authors:** Rachel A. Prusynski, Zhiyu Yan, Kori S. Zachrison, Tracy M. Mroz, Renee Y. Hsia, Amber K. Sabbatini

**Affiliations:** 1 Department of Rehabilitation Medicine, School of Medicine, University of Washington, Seattle; 2 Department of Health Systems and Population Health, School of Public Health, University of Washington, Seattle; 3 Department of Biostatistics, Harvard T. H. Chan School of Public Health, Boston, Massachusetts; 4 Department of Emergency Medicine, Harvard Medical School, Boston, Massachusetts; 5 Department of Emergency Medicine, Massachusetts General Hospital, Boston; 6 Philip R. Lee Institute for Health Policy Studies, University of California, San Francisco; 7 Department of Emergency Medicine, University of California, San Francisco; 8 Department of Emergency Medicine, School of Medicine, University of Washington, Seattle

## Abstract

**Importance:**

Delays in hospital discharge to skilled nursing facilities (SNFs) are widely viewed by hospital administrators and policymakers as a significant contributor to hospital crowding and adverse patient outcomes. However, the extent to which regional SNF capacity is associated with hospital length of stay (LOS) is not well understood.

**Objectives:**

To construct empirically derived hospital-SNF markets using a network science approach and to examine whether variation in SNF capacity within these markets is associated with hospital LOS.

**Design, Setting, and Participants:**

This cross-sectional study used 2018 and 2019 Medicare fee-for-service claims linked with publicly available SNF data and included 3.34 million inpatient admissions discharged to SNFs from short-term hospitals in the US. Data were analyzed from October 1, 2024, to February 28, 2026.

**Exposures:**

Monthly mean SNF occupancy rates and nurse and therapy staffing levels within an empirically defined regional hospital-SNF market from a patient-sharing network analysis.

**Main Outcomes and Measures:**

The main outcome was hospital LOS. Linear regression models estimated associations between market-level SNF capacity measures and LOS, adjusting for patient characteristics, hospital-level postacute care demand, SNF organizational features, and time and hospital-SNF market fixed effects. Analyses were stratified by dual Medicare-Medicaid eligibility.

**Results:**

The study sample included 3.34 million Medicare inpatient admissions (mean [SD] age, 78.6 [11.3] years; 60.2% women) discharged to SNFs across 62 303 hospital-SNF dyads, and 421 empirically derived hospital-SNF markets. The mean (SD) hospital LOS was 6.9 (6.8) days (7.4 [7.8] days for dual-eligible patients and 6.7 [6.2] days for non–dual-eligible patients). Within markets, each additional nurse staffing hour per patient-day was associated with a 3.5% shorter LOS (95% CI, −5.5% to −1.4%); associations were also found among dual-eligible patients (–3.9% [95% CI, –6.7% to –0.9%) and non–dual-eligible patients (–3.2% [95% CI, –5.4% to –0.9%]). Differences in occupancy rates and therapy staffing within networks were not associated with differences in LOS after adjusting for patient, hospital, and network factors.

**Conclusions and Relevance:**

Hospital-SNF markets defined by the strength of patient-sharing relationships provide a reproducible framework for examining postacute care capacity across payer populations. Within these markets, for the Medicare fee-for-service population, higher nurse staffing levels were associated with shorter hospital LOS. Strategies to reduce discharge delays and hospital crowding may require closer alignment between hospitals and highly connected SNFs to maintain adequate postacute care capacity.

## Key Points

**Question:**

Is skilled nursing facility (SNF) capacity within hospital-SNF markets associated with hospital length of stay?

**Findings:**

This cross-sectional study of 3.34 million Medicare fee-for-service hospitalizations in 2018 and 2019 identified 421 hospital-SNF markets using community detection. Within markets, lower nurse staffing levels were associated with longer hospital stays, particularly among beneficiaries dually eligible for Medicare and Medicaid.

**Meaning:**

These findings suggest that network-defined hospital-SNF markets provide a reproducible framework for measuring postacute care capacity and may inform strategies to address hospital discharge delays.

## Teaser

This cross-sectional study examines whether variation in regional skilled nursing facility capacity is associated with hospital length of stay.

## Introduction

Delayed discharge from the hospital contributes to reduced inpatient capacity and boarding of inpatients in the emergency department. Compared with commercially insured patients, patients with Medicare are 1.5 times more likely and patients dually eligible for Medicare and Medicaid are 2.5 times more likely to experience avoidable discharge delays.^1^ Discharge delays for patients requiring postacute care, including skilled nursing facility (SNF) care, have been shown to result in longer hospital length of stay (LOS).^2,3^ Between 2019 and 2022, hospital LOS for patients discharged to postacute care increased by 24%, compared with a 19.2% increase for all hospitalized patients.^4^

Constrained SNF capacity may exacerbate discharge delays from the hospital and limit access to postacute care. SNF capacity has decreased since the COVID-19 pandemic,^5^ and the Medicare Payment Advisory Commission specifically cited staffing challenges in SNFs as a potential contributor to hospital crowding.^2,6,7^ However, while efforts to support the SNF workforce and avoid SNF closures are ongoing,^8,9,10,11^ it is unclear whether policies aimed at improving or maintaining SNF capacity and staffing will have upstream benefits in reducing hospital LOS.

To date, few studies have examined the extent to which SNF capacity and organizational characteristics shape upstream hospital outcomes, such as LOS. Moreover, no standard method exists to define which SNFs are most relevant when measuring hospital-level effects of constrained SNF capacity. For example, insufficient staffing and high occupancy in SNFs that typically admit patients from a given hospital would be expected to have a larger association with crowding than SNFs to which the hospital infrequently refers patients. In addition, hospitals may be competing for SNF beds, such that constrained SNF capacity may have upstream effects on multiple hospitals. Prior work does not model the system-level, interdependent associations of hospitals and SNFs within a market that accounts for the strength of their patient-sharing relationships.

Although 1 study examined hospital-SNF connections as measured by hospital ownership of SNFs, number of SNFs to which a hospital discharges at least 5 patients, and concentration of a hospital’s discharges to SNFs, the data are now outdated and the study did not consider patient outcomes.^12^ Other prior work has compared SNF market definitions based on geographic boundaries (eg, county, fixed distances around an SNF), patient flows, market concentrations,^13^ and defined rehabilitation service areas based on utilization of SNFs, inpatient rehabilitation facilities, long-term care hospitals, and home health care,^14^ but neither of these studies connected SNFs with a particular hospital. Finally, geographic measures that define health care markets such as hospital referral regions and hospital service areas, commonly used in health services research, are inadequate for examining associations between SNF capacity and hospital LOS because they capture only hospital utilization and do not account for the strength of the association between individual hospitals and SNFs or for the potentially simultaneous effects of constrained SNF capacity on multiple hospitals.^5,15^

To address these gaps, we used network-based methods to examine whether regional SNF capacity is associated with hospital LOS among Medicare fee-for-service (FFS) beneficiaries within empirically derived hospital-SNF markets. We further examined whether these associations between regional SNF capacity and hospital LOS vary by dual-eligibility status. We hypothesized that as SNF capacity decreased in a market, LOS for patients discharged to an SNF in that same market would also increase.

## Methods

### Dataset and Study Population

This study was a retrospective cross-sectional analysis of Medicare Provider Analysis and Review (MedPAR) files from 2018 and 2019. We included all adult inpatient hospitalizations for beneficiaries discharged to an SNF, defined as the presence of an SNF admission within 3 days of the hospital discharge date. We excluded data from the COVID-19 pandemic due to documented severe disruptions in postacute care capacity and access. We excluded beneficiaries who were discharged against medical advice, those who had a recorded discharge disposition of SNF but did not have an identifiable SNF admission within 3 days, children younger than 18 years, and individuals who were not enrolled in Medicare for at least 3 months after hospital discharge.

This study was deemed exempt from ethics review and the need for informed consent by the University of Washington Institutional Review Board because this was secondary research of data originally collected and generated by the government for nonresearch purposes and subject to a data use agreement with the Centers for Medicare & Medicaid Services (CMS). The study followed the Strengthening the Reporting of Observational Studies in Epidemiology (STROBE) reporting guideline.

### Market Creation

We used a network science approach to characterize markets of strongly connected hospitals and SNFs based on the number of shared patients with Medicare FFS between facilities during our 2018-2019 study period.^16,17^ We then identified individual hospitals and SNFs using Medicare provider numbers and limited our sample to the hospital-SNF dyads that shared at least 2 patients in both 2018 and 2019. We chose a minimum threshold of 2 shared patients to enter the network model to avoid identification of spurious associations while still ensuring we included smaller-volume sites. However, this does not mean that hospital-SNF dyads with minimal patient sharing will be grouped together in the same market, as our network model specifically accounted for the volume of shared patients in defining markets. We constructed a bipartite network graph including nodes of hospitals and SNFs. Edges between hospital and SNF nodes were based on dyads with shared patients as above. Edges were weighted using the total number of shared patients per dyad in 2018 and 2019. We used community detection via the Leiden algorithm to identify clusters of hospitals and SNFs more closely connected to each other than to nodes in other clusters.^18,19^ The algorithm maximizes a bipartite extension of modularity and assigns each facility to a single cluster.^20^ This results in clusters representing markets of closely related facilities, and limits an individual hospital or SNF to a single market. Further details on the network science and community detection methods are provided in the eAppendix in Supplement 1.

### SNF Capacity Variables

We used public data sources on SNF organizational characteristics, volume, and staffing from the same years to create variables reflecting SNFs’ capacity to admit patients with Medicare FFS within each market. We calculated occupancy rates by dividing daily patient census from CMS Payroll Based Journal files by the certified bed count from CMS Provider of Services files and then mean daily occupancy rates for each month of the study period. For measures of capacity based on staffing, we used the Payroll Based Journal data and divided daily staffing hours for nursing staff (registered nurses, licensed practical nurses, and certified nursing assistants) and therapy staff (physical and occupational therapists and assistants and speech language pathologists) by daily patient census. Mean daily staffing levels were calculated at the SNF-month level once we removed highly improbable staffing values per CMS methods.^21^ Once monthly capacity measures were calculated for each SNF, we multiplied each measure by the proportion of total beds in the hospital-SNF market each SNF contributed, such that capacity for larger SNFs received more weight, before averaging across all SNFs in the cluster to create market-level monthly mean SNF capacity.

### SNF Organizational Characteristics

We included covariates reflecting organizational characteristics of SNFs within markets. From quarterly CMS Provider of Services files, we included whether the SNF was hospital-based vs freestanding, ownership (for profit, not for profit, government), and an indicator if the SNF changed ownership in the prior year. We used LTCFocus files^22^ to calculate the proportion of all patients (both postacute care and long-term care) within an SNF whose primary payer was Medicaid as a measure of beds available for Medicare beneficiaries. Organizational characteristics were then averaged across all SNFs in the market to create network-level variables.

### Patient and Hospital Stay Characteristics

Risk adjustment characteristics included age, sex, weighted Elixhauser Comorbidity Index, and indicators for end-stage kidney disease and Alzheimer disease or other dementia diagnosis. Hospital stay characteristics included major diagnostic category of the primary hospital diagnosis and indicators for intensive care unit stay, surgical procedure, and Medicare bundled payment participation. In addition, to capture postacute care demand at the hospital level, we calculated the monthly proportion of patients who discharged to a postacute setting (ie, SNF, home health, inpatient rehabilitation, or long-term acute care hospital) out of all patients with Medicare FFS who were discharged alive and did not leave against medical advice.

### Statistical Analysis

Statistical analysis was conducted from October 1, 2024, to February 28, 2026. Monthly SNF capacity and organizational variables at the market level were matched with hospital claims using a crosswalk of Medicare provider numbers for facilities in each hospital-SNF market identified in our network analysis. Specifically, we used the date of hospital discharge and hospital provider numbers to merge MedPAR hospital claims with monthly SNF capacity and organizational measures within that hospital’s market for each encounter. Then, we estimated associations between our outcome of hospital LOS and measures of SNF capacity within the hospital’s market using linear regression. The 3 SNF capacity measures (occupancy and nurse and therapist staffing levels) were modeled together in a single regression. Because LOS is highly right skewed, we used the natural log of LOS as the outcome to stabilize the residual variance and reduce the influence of extreme values.

Our models adjusted for patient-level and hospital stay–level characteristics and SNF organizational characteristics as described above. As SNF characteristics were often time varying, we included fixed effects for chronological month of our study (ie, 1-24 months). We included hospital-SNF market fixed effects such that we were comparing periods of higher vs lower capacity constraints within each individual market. Finally, we used cluster-robust standard errors to account for correlation in outcomes within hospitals and within hospital-SNF markets.^23^ Given the greater likelihood of avoidable delays in discharge experienced by beneficiaries dually eligible for Medicare and Medicaid,^1^ we conducted stratified analyses for dual-eligible and non–dual-eligible beneficiaries separately. We also conducted sensitivity analyses by lagging the occupancy rate and staffing level exposures by 1 month to test whether SNF capacity within markets in the month prior to the time of hospital discharge were associated with hospital LOS.

Analyses were conducted with R, version 4.5.0 (R Project for Statistical Computing). Networks were constructed using the igraph package, version 2.0.3. Community detection was performed using the leiden R package, version 0.4.3.1, which interfaces with the Python leidenalg library, version 0.10.2 (Python Software Foundation), and implements bipartite modularity optimization via the ModularityVertexPartition.Bipartite method. Two-sided *P* < .05 was considered statistically significant using linear regression with cluster-robust standard errors.

## Results

### Hospital-SNF Markets

We identified 62 303 hospital-SNF dyads between 4148 hospitals and 14 714 SNFs in 2018 and 2019. Hospitals were connected with 1 to 188 SNFs (median, 8 SNFs [IQR, 3-20 SNFs]) through patient transfer and SNFs received patients from 1 to 23 hospitals (median, 4 hospitals [IQR, 2-5 hospitals]). This varied by rural vs urban location (as indicated in Provider of Services files), with more connections among urban hospitals. Urban hospitals had a median of 15 connected SNFs (IQR, 6-28 SNFs) vs only 3 connected SNFs (IQR, 2-6 SNFs) for rural hospitals. Urban SNFs had a median of 4 connected hospitals (IQR, 3-6 hospitals) vs 3 connected hospitals (IQR, 2-5 hospitals) for rural SNFs.

**Figure 1.**
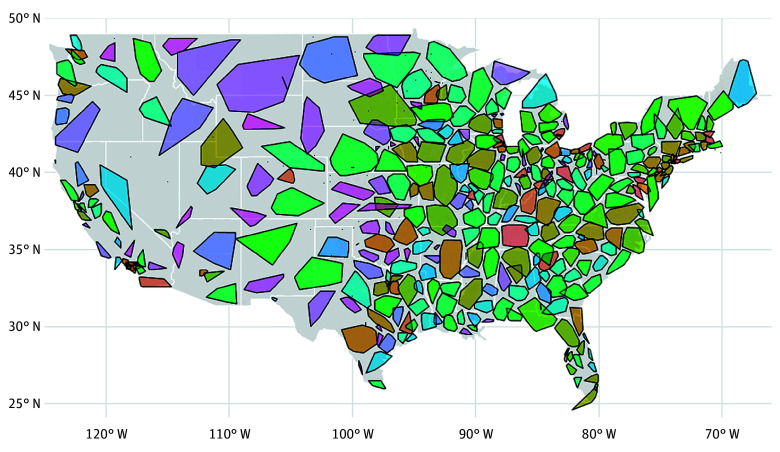
Map Showing Location and Geographic Expanse of Hospital–Skilled Nursing Facility (SNF) Markets Outlined Using Community Detection The colors in the map delineate the separate markets. Markets in Alaska (n = 1) and Hawaiʻi (n = 1) are not shown for ease of visualization.

Of the 438 markets of closely related hospitals and SNFs identified through our community detection approach, we excluded 17 that included only a single facility, giving a final sample of 421 markets (Table 1). Each market included a median of 10 hospitals (IQR, 5-14 hospitals), 40 SNFs (IQR, 19-77 SNFs), and 96 referral dyads (IQR, 37-210 dyads). On average, each dyad shared a median of 46.2 patients (IQR, 33.8-62.2 patients) within a market. Table 1 provides information on more characteristics of these markets, including facility bed count, patient volume, facility rurality profile, and geographic expanse calculated from facility zip codes. The geographic expanse and location of each hospital-SNF market are displayed on a map of the continental US in Figure 1.

**Table 1.** Characteristics of Hospital-SNF Markets (N = 421) Containing 4148 Hospitals and 14 714 SNFs

Market characteristic	Median (IQR) value
Network size	
No. of hospitals	10 (5-14)
No. of SNFs	40 (19-77)
No. of hospital-SNF dyads	96 (37-210)
Hospital bed count	250.2 (161.6-387.3)
SNF bed count	103.9 (86.4-118.3)
Total No. of patients in 2018 and 2019	4831 (2004-10 355)
Shared patients per dyad in 2018 and 2019	46.2 (33.8-62.2)
Geographic reach	
No. of hospital counties	4 (2-8)
No. of SNF counties	11 (4-18)
No. of hospital states	1 (1-1)
No. of SNF states	1 (1-2)
Mean geographic expanse between hospital-SNF dyads, miles^a^	13.8 (7.2-20.9)
Mean % of rural hospitals	15.3 (0.8-45.0)
Mean % of rural SNFs	34.4 (6.4-73.1)

Abbreviation: SNF, skilled nursing facility.

^a^ To convert miles to kilometers, multiply by 1.6.

### Associations Between SNF Capacity and Hospital LOS

We included 3 341 487 hospitalizations (mean [SD] age, 78.6 [11.3] years; 60.2% women and 39.8% men) resulting in an SNF stay that had complete data on hospital-SNF market characteristics (Table 2). eTable 1 in Supplement 1 provides a flowsheet of missing SNF-hospital network data. Of those hospitalizations, 1 101 439 (33.0%) were for dual-eligible FFS beneficiaries (Table 2). The mean (SD) LOS for all hospital stays was 6.9 (6.8) days; the mean (SD) LOS was longer for dual-eligible beneficiaries than for non–dual-eligible beneficiaries (7.4 [7.8] vs 6.7 [6.2] days). Across all hospital stays in each market, the monthly SNF capacity measures weighted for SNF size were as follows: the mean (SD) SNF occupancy rate was 81.1% (8.1%), nurse staffing levels were a mean (SD) of 3.4 (0.3) hours per patient-day, and therapy staffing levels were a mean (SD) of 0.4 (0.1) hours per patient-day.

**Table 2.** Hospital Stay and Patient Characteristics for 3 341 487 Medicare Fee-for-Service Beneficiaries Discharged From a Hospital to an SNF and the Mean Monthly SNF Capacity and Organizational Variables Per Hospital Stay

Characteristic	Value
Patient and hospital stay characteristics	
Length of stay, mean (SD), d	6.9 (6.8)
Dually eligible for Medicare and Medicaid, No. (%)	1 101 439 (33.0)
Female sex, No. (%)	2 011 080 (60.2)
Male sex, No. (%)	1 330 407 (39.8)
Age, mean (SD), y	78.6 (11.3)
Intensive care unit stay, No. (%)	1 054 248 (31.6)
Surgical procedure, No. (%)	1 937 954 (58.0)
Bundled claim, No. (%)	579 024 (17.3)
Top 5 major diagnostic categories for primary diagnosis	
Disease and disorders of the musculoskeletal system and connective tissue, No. (%)	748 375 (22.4)
Disease and disorders of the circulatory system, No. (%)	490 335 (14.7)
Infectious and parasitic diseases, No. (%)	416 612 (12.5)
Diseases and disorders of the respiratory system, No. (%)	355 981 (10.7)
Diseases and disorders of the kidney and urinary tract, No. (%)	311 542 (9.3)
Elixhauser Comorbidity Index, mean (SD)	12.0 (9.2)
End-stage kidney disease, No. (%)	181 553 (5.4)
Alzheimer or dementia diagnosis, No. (%)	132 281 (4.0)
Admitted patients discharged to postacute care, mean (SD), %	46.0 (9.5)
Market-level SNF capacity variables^a^	
SNF occupancy rate, mean (SD), %	81.1 (8.1)
Nursing staff hours per patient-day, mean (SD)	3.4 (0.3)
Therapy staffing hours per patient-day, mean (SD)	0.4 (0.1)
Market-level SNF organizational characteristics	
SNFs connected to hospital, mean (SD), No.	35.7 (30.5)
For-profit SNFs, mean (SD), %	74.6 (13.8)
Hospital-based SNFs, mean (SD), %	2.4 (3.0)
Chain SNFs, mean (SD), %	59.7 (18.9)
SNFs with recent change in ownership, mean (SD), %	6.5 (6.9)
Medicaid patients, mean (SD), %	56.7 (7.3)

Abbreviation: SNF, skilled nursing facility.

^a^ SNF capacity variables are weighted by the proportion of beds each SNF contributes to the market, averaged monthly within each hospital-SNF market, and then linked with hospital claims based on the month of hospital discharge and hospital Medicare provider number. Other organizational variables were averaged monthly but were not weighted. Descriptive statistics for market variables were then calculated across all 3 341 487 hospital stays.

**Figure 2.**
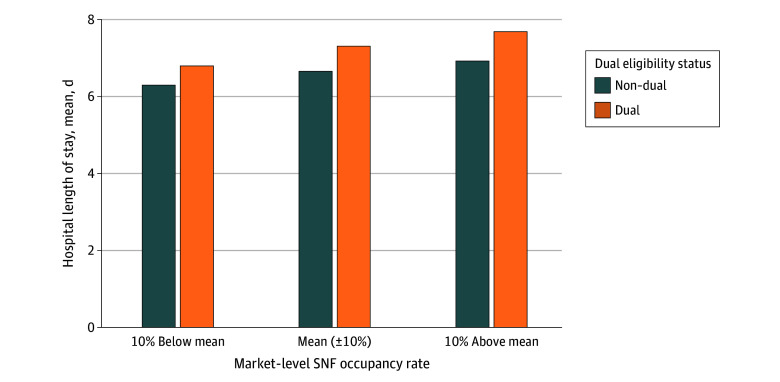
Bar Graph Showing Unadjusted Mean Hospital Length of Stay for 3 341 487 Dual-Eligible and Non–Dual-Eligible Fee-for-Service Beneficiaries Hospital stays are grouped based on whether the monthly occupancy rates across skilled nursing facilities (SNFs) in their hospital-SNF markets were within 10% of the mean, at least 10% below the mean, or at least 10% above the mean.

Figure 2 shows unadjusted LOS for dual-eligible and non–dual-eligible beneficiaries separated by the SNF occupancy rates relative to the mean across all hospital stays. Coefficients for the adjusted associations between hospital LOS and SNF capacity variables within the hospital-SNF markets are provided in Table 3. Within a given hospital-SNF network, periods of higher SNF occupancy were associated with slightly longer hospital LOS, but differences were not statistically significant once adjusted for patient characteristics and network and time fixed effects and clustered standard errors. One additional hour of mean nurse staffing per patient-day among SNFs in each market was associated with a 3.5% shorter hospital LOS (95% CI, −5.5% to −1.4%) for all FFS beneficiaries. Higher nurse staffing of 1 hour per patient-day was associated with a 3.9% shorter hospital LOS (95% CI, −6.7% to −0.9%) for dual-eligible beneficiaries and a 3.2% shorter hospital LOS (95% CI, −5.4% to −0.9%) for non–dual-eligible beneficiaries. Within each hospital-SNF market, differences in therapy staffing levels were not associated with statistically significant differences in hospital LOS. Results of a sensitivity analysis using lagged capacity variables were consistent with primary analyses (eTable 2 in Supplement 1).

**Table 3.** Associations Between SNF Capacity Variables Within Hospital-SNF Markets and the Hospital Length of Stay for Medicare Fee-for-Service Beneficiaries Discharged From a Hospital to an SNF in 2018 and 2019

Variable	Percent change in length of stay (95% CI), %		
	All beneficiaries (N = 3 341 487)	Dual-eligible beneficiaries (n = 1 101 439)	Non–dual-eligible beneficiaries (n = 2 240 048)
Market SNF capacity variables^a^			
SNF occupancy rate (10-point increase)	0.4 (−1.0 to 1.7)	0.5 (−1.4 to 2.4)	0.4 (−1.0 to 1.7)
Nursing staff hours per patient-day (1-h increase)	−3.5 (−5.5 to −1.4)	−3.9 (−6.7 to −0.9)	−3.2 (−5.4 to −0.9)
Therapy staffing hours per patient-day (15-min increase)	0.2 (−1.4 to 1.8)	−0.1 (−2.1 to 2.0)	0.3 (−1.3 to 1.9)
Market SNF organizational covariates			
SNFs with recent changes in ownership (10-point increase)	−2.1 (−4.8 to 0.7)	−1.3 (−6.0 to 3.8)	−2.2 (−5.5 to 1.3)
For-profit SNFs (10-point increase)	0.5 (−2.5 to 3.5)	3.0 (−0.6 to 6.7)	−0.9 (−4.1 to 2.5)
Hospital-based SNFs (1-point increase)	−0.2 (−0.7 to 0.3)	−0.4 (−1.1 to 0.3)	−0.02 (−0.6 to 0.5)
Chain SNFs (10-point increase)	1.0 (−1.2 to 3.2)	−0.3 (−3.4 to 2.9)	1.6 (−1.1 to 4.2)
Medicaid patients (10-point increase)	1.3 (0.1 to 2.6)	0.9 (−0.6 to 2.4)	1.7 (0.3 to 3.1)

Abbreviation: SNF, skilled nursing facility.

^a^ SNF capacity variables are weighted by the proportion of beds each SNF contributes to the market and then averaged across all SNFs in each discharging hospital’s market. Linear regression models were adjusted for all patient and hospital stay characteristics in Table 2 as well as study month and hospital-SNF market fixed effects. Standard errors account for clustering at the hospital and network levels.

## Discussion

In this study of Medicare FFS beneficiaries, we used network science methods to identify 421 networks of closely connected hospitals and SNFs across the US based on the number of shared patients with Medicare FFS. Empirically defining these networks allows us to acknowledge how hospitals and SNFs exist in an interdependent system where the volume of transfers and case-mix of all units in the network may have simultaneous associations with other units. Although only FFS data were available for our analyses, these network science methods provide a framework to reproduce similar analyses with all-payer data to better reflect patient-sharing relationships for all patients discharging from hospitals to SNFs, which will be especially important considering the larger increase in hospital LOS for the growing Medicare Advantage population, compared with FFS, in recent years.^3^ Within these empirically derived markets, our analysis examining the association of SNF capacity and hospital LOS found that lower rates of nurse staffing had a measurable upstream association with longer inpatient hospitalizations. Specifically, periods where nurse staffing decreased by 1 hour per patient-day among SNFs in the discharging hospital’s market were associated with 3.5% longer hospital LOS, translating to roughly 6 additional hours of excess LOS. We observed slightly larger effects for dual-eligible beneficiaries compared with non–dual-eligible beneficiaries.

The associations between measures of constrained SNF capacity in the market and longer hospital LOS may have meaningful implications at the health system level. Even a small percentage increase in LOS, when applied to the more than 3 million Medicare beneficiaries discharged to an SNF annually, may translate into substantial operational challenges and increased bed-days for hospitals facing crowding. However, several factors may also attenuate the associations we have identified here. First, the study population was limited to Medicare FFS beneficiaries, who are often prioritized for SNF admission because of relatively favorable reimbursement, making this population less sensitive to changes in SNF capacity. When SNF capacity becomes more strained, SNFs may preferentially accept Medicare FFS patients over patients with other payers with lower reimbursement and preauthorization requirements, including commercial, Medicare Advantage, and Medicaid managed care patients.^24,25,26^ Second, beyond the magnitude of the estimated associations, this study introduces a reproducible network-based framework for defining hospital–SNF markets and linking time-varying postacute care capacity with upstream hospital outcomes. Demonstrating associations within this framework provides proof of concept for future all-payer analyses that may reveal larger system-level effects.

In addition to measuring network associations, this is the first study, to our knowledge, to include market-level measures of SNF capacity that reflect the availability of SNF beds alongside the availability of nursing and therapy staff to care for admitted SNF patients. Staffing levels are considered a key component of SNF quality,^21,27^ but SNFs are experiencing unprecedented staffing challenges in the wake of the COVID-19 pandemic,^5,6,7,28,29,30^ highlighting the need to incorporate staffing into measurement of SNF capacity and its association with hospital crowding. We also accounted for SNF organizational characteristics, such as recent ownership changes, Medicaid payer mix, and profit status, that may vary over time and impact individual SNFs’ willingness or ability to admit FFS patients.^31,32,33^

Despite unadjusted trends of longer hospital LOS in networks with higher SNF occupancy (Figure 2), after accounting for hospital-SNF market fixed effects, we did not observe an association between SNF occupancy rates and hospital LOS. One potential explanation is that the Medicare FFS population included in this study remains relatively desirable to SNFs even during periods of high occupancy. Prior work has found that SNFs engage in selective admission practices based on payer,^3,24,26,34^ often prioritizing FFS patients due to higher postacute reimbursement compared with Medicaid and Medicare Advantage contracts.^3,6,25,26,35^ As a result, even during periods of high occupancy, SNFs within established hospital-SNF networks may continue to prioritize FFS admissions, whereas nurse staffing levels may better capture true operational constraints that are associated with admission throughput, even for the desirable FFS population.

Our results demonstrated a larger relative increase in hospital LOS in markets with more constrained SNF capacity—measured by lower nurse staffing levels—for dual-eligible beneficiaries compared with non–dual-eligible beneficiaries. These results are consistent with reports of more substantial discharge delays for dual-eligible beneficiaries^1^ and consistent with the longstanding issue of SNFs deprioritizing patients with higher perceived social and financial risks, which can include dual-eligible patients with low income.^24,26,34,36^ These results are pertinent for hospitals serving higher proportions of dual-eligible beneficiaries, as these hospitals may experience greater issues with delayed discharges to SNFs in their markets as those SNFs face increasing staffing limitations.

### Limitations

This study has some limitations. Our community detection approach created hospital-SNF markets based on the number of shared Medicare FFS patients in 2018 and 2019. As hospital discharge practices and SNF capacity constraints shifted during the COVID-19 pandemic, these networks may have reorganized, and markets must be redefined if analyses are conducted on more recent data. Markets were identified based on patterns of care and sharing of patients with FFS Medicare only. Thus, these networks are not generalizable to patients with Medicare Advantage, commercial insurance, or Medicaid-only coverage if discharge practices or patient sharing patterns vary by payer. We set our parameters to ensure each facility was assigned to only a single market for ease of matching SNF capacity variables with hospital stays based on market membership. In practice, hospitals and SNFs may participate in multiple overlapping referral patterns. Future work could explore soft or probabilistic clustering methods to account for such shared affiliations. SNF capacity measures were taken from public data that allowed us to create time-varying measures of SNF capacity. However, staffing and census data in the Payroll Based Journal are subject to some limitations, including potential underreporting for salaried staff, lags in quarterly certified bed counts, and other potential reporting errors. Finally, time-varying capacity constraints in postacute care markets may change which patients are referred to an SNF, and this selection is not fully accounted for in the present analysis.

## Conclusions

In this national cross-sectional study of Medicare inpatient stays, we empirically identified 421 markets containing closely connected hospitals and SNFs based on patient sharing. When exploring how hospital LOS varied during times of more constrained SNF capacity within each market, we found that periods of lower nurse staffing in SNFs were associated with slightly longer hospital LOS. Associations between constrained SNF capacity and longer LOS were slightly larger for dual-eligible patients compared with non–dual-eligible patients. For hospitals seeking to reduce potential discharge delays and address hospital crowding associated with SNF capacity, these results highlight the need to form new partnerships or strengthen existing partnerships with network SNFs and promote efforts to support SNF capacity.

## Notes

Supplement 1. **eAppendix.** Network Science and Community Detection Approaches
eReferences
**eFigure.** Illustration of Bipartite Community Detection to Identify Hospital-SNF Markets Within the Hospital-SNF Transfer Network **eTable 1.** Flowsheet for Missing SNF-Hospital Network Data **eTable 2.** Results of Sensitivity Analysis With SNF Capacity Variables Lagged by One Month

Supplement 2.
Data Sharing Statement
