# Supplementary material for: Skilled Nursing Facility Network Capacity and Hospital Length of Stay
Source: JAMA Netw Open. 2026 Apr 30;9(4):e269930. doi: 10.1001/jamanetworkopen.2026.9930 (PMC13133692; doi:10.1001/jamanetworkopen.2026.9930)
Supplement: Supplement 1. — eAppendix. Network Science and Community Detection Approaches eReferences eFigure. Illustration of Bipartite Community Detection to Identify Hospital-SNF Markets Within the Hospital-SNF Transfer Network eTable 1. Flowsheet for Missing SNF-Hospital Network Data eTable 2. Results of Sensitivity Analysis With SNF Capacity Variables Lagged by One Month [file jamanetwopen-e269930-s001.pdf]

Supplementary Online Content

Prusynski RA, Yan Z, Zachrison KS, Mroz TM, Hsia RY, Sabbatini AK. Skilled nursing facility network capacity and hospital length of stay. *JAMA Netw Open.* 2026;9(4):e269930. doi:10.1001/jamanetworkopen.2026.9930

eAppendix. Network Science and Community Detection Approaches

eReferences

eFigure. Illustration of Bipartite Community Detection to Identify Hospital-SNF Markets Within the Hospital-SNF Transfer Network

eTable 1. Flowsheet for Missing SNF-Hospital Network Data

eTable 2. Results of Sensitivity Analysis With SNF Capacity Variables Lagged by One Month

This supplementary material has been provided by the authors to give readers additional information about their work.

eAppendix. Network Science and Community Detection Approaches

Network science approaches have been applied to map patient care delivery patterns, access to care, and variation between markets in the acute care setting for a variety of conditions. This includes critical care, emergency general surgery, acute myocardial infarction and stroke.[1–4] This prior work has used interhospital transfer of patients to identify connections between hospitals and groups of hospitals that are closely connected in the care of patients. We extend this work to the post-acute setting to identify patient flow between hospitals and skilled nursing facilities (SNFs). Where prior work based on the hospital setting has generated unipartite network graphs – i.e., all nodes in the network were of the same type (hospitals), in our case we generated a bipartite network graph composed of two different types of nodes: hospitals and SNFs. An illustration of this process is included in eFigure 1.

We constructed the hospital-SNF transfer network by identifying nodes (hospitals and SNFs) connected through patient transfer. We required at least two patient transfers between any given hospital and SNF pair to avoid the inclusion of spurious connections. Connected pairs (i.e., dyads) were used to generate the hospital-SNF transfer network. We examined global and local network characteristics for face validity (e.g., total number of transfers, mean number of transfers between a hospital-SNF dyad). Networks were generated using the *igraph* R package (version 2.0.3).

We next sought to identify clusters of hospitals and SNFs that are closely connected through patients transfers for the identification of hospital-SNF markets. The network science approach for identifying such clusters is broadly termed community detection. Because our hospital-SNF network is bipartite by construction, communities were identified by maximizing weighted bipartite modularity. The bipartite modularity measures the extent to which the total transfer volume within a proposed hospital-SNF community is greater than would be expected under a bipartite null model that preserves each node's overall transfer volume (i.e., hospitals that discharge more and SNFs that receive more are expected to have more connections even in the null model).[5] Edges were weighted by the number of shared patient transfers between each hospital-SNF dyad, such that higher-volume referral relationships contributed proportionally more to the objective modularity function to be maximized.

While a number of community detection approaches have been developed, we chose to use the Leiden algorithm given its ready application to a bipartite network and its ability to cleanly partition nodes into distinct clusters.[6,7] The Leiden algorithm identifies the communities by iteratively optimizing the objective function (weighted bipartite modularity in our case). The algorithm proceeds in three phases: (1) local movement of nodes to

© 2026 Prusynski RA et al. *JAMA Network Open.*

neighboring communities to improve the objective function, (2) refinement of communities to ensure they are internally well connected, and (3) aggregation of the network into super-nodes followed by repetition of the procedure until no further improvement is possible. Compared with the widely used Louvain algorithm, Leiden improves partition quality by preventing poorly connected or disconnected communities and providing stronger guarantees of internal connectivity and convergence. We implemented this approach using the *leiden* R package (version 0.4.3.1), which interfaces with the Python *leidenalg* library (version 0.10.2), specifying the *ModularityVertexPartition.Bipartite* method. The resolution parameter of 0.01 was selected to control community granularity such that the number of resulting communities is roughly on the same scale as the number of existing metropolitan statistical areas (387).[8] This strategy enabled the identification of distinct communities of hospitals and SNFs into what we termed hospital-SNF networks.

© 2026 Prusynski RA et al. *JAMA Network Open.*

eReferences

1. Zachrison KS, Hsia RY, Schwamm LH, et al. Insurance-Based Disparities in Stroke Center Access in California: A Network Science Approach. *Circ Cardiovasc Qual Outcomes*. 2023;16(10). doi:10.1161/CIRCOUTCOMES.122.009868

2. Iwashyna TJ, Christie JD, Moody J, Kahn JM, Asch DA. The Structure of Critical Care Transfer Networks. *Med Care*. 2009;47(7).

3. Iwashyna TJ, Kahn JM, Hayward RA, Nallamothu BK. Interhospital Transfers Among Medicare Beneficiaries Admitted for Acute Myocardial Infarction at Nonrevascularization Hospitals. *Circ Cardiovasc Qual Outcomes*. 2010;3(5):468-475. doi:10.1161/CIRCOUTCOMES.110.957993

4. Teng CY, Davis BS, Rosengart MR, Carley KM, Kahn JM. Assessment of Hospital Characteristics and Interhospital Transfer Patterns of Adults With Emergency General Surgery Conditions. *JAMA Netw Open*. 2021;4(9):e2123389. doi:10.1001/jamanetworkopen.2021.23389

5. Barber MJ. Modularity and community detection in bipartite networks. *Phys Rev E*. 2007;76(6):066102. doi:10.1103/PhysRevE.76.066102

6. Hairol Anuar SH, Abas ZA, Yunos NM, et al. Comparison between Louvain and Leiden Algorithm for Network Structure: A Review. *J Phys Conf Ser*. 2021;2129(1):012028. doi:10.1088/1742-6596/2129/1/012028

7. Traag VA, Waltman L, Van Eck NJ. From Louvain to Leiden: guaranteeing well-connected communities. *Sci Rep*. 2019;9(1):5233. doi:10.1038/s41598-019-41695-z

8. United States Census Bureau. About Metropolitan and Micropolitan Statistical Areas. July 2023. Accessed September 17, 2025. https://www.census.gov/programs-surveys/metro-micro/about.html

© 2026 Prusynski RA et al. *JAMA Network Open.*

eFigure. Illustration of Bipartite Community Detection to Identify Hospital-SNF Markets Within the Hospital-SNF Transfer Network

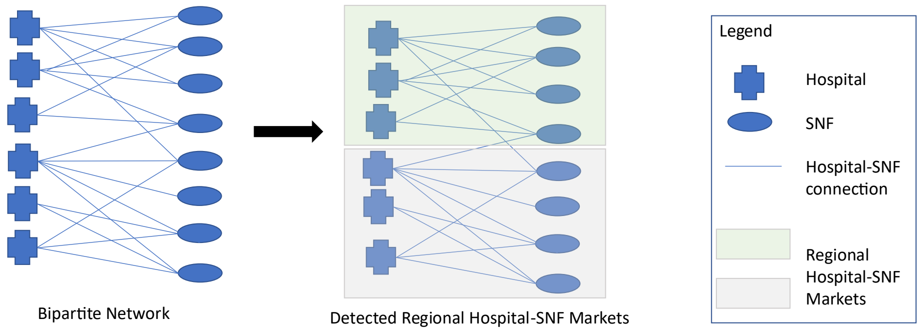

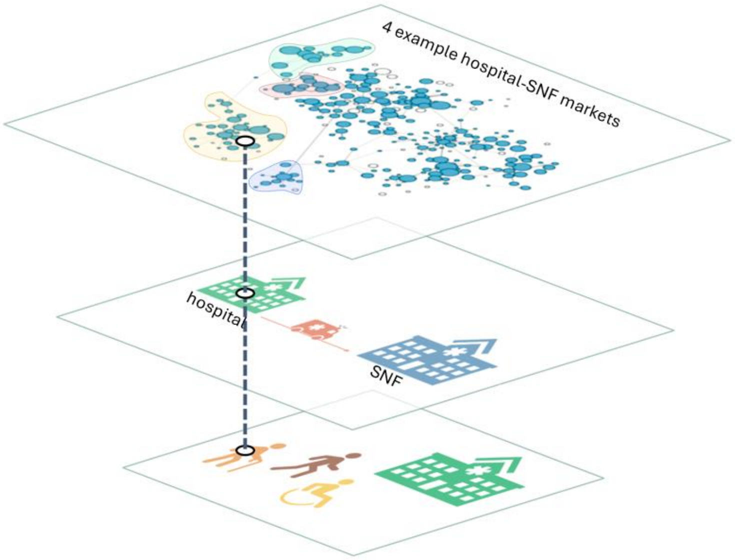

© 2026 Prusynski RA et al. *JAMA Network Open.*

eTable 1. Flowsheet for Missing SNF-Hospital Network Data

Sample Description	Sample Size	N (%) Missing
Medicare fee-for-service hospitalizations ending in SNF stay in 2018 and 2019 meeting the following inclusion criteria: verifiable enrollment in Medicare for 3 months after hospital stay, did not discharge against medical advice.	3,342,648	N/A
Skilled nursing facilities in the hospital's network reported data on census and staffing from Payroll Based Journal during month of hospitalization.	3,342,458	190 (0.0%)
Skilled nursing facilities in the hospital's network reported data on ownership status, changes in ownership, and bed count in Provider of Services File.	3,342,458	0 (0.0%)
Skilled nursing facilities (SNFs) in the hospital's network had data on payer mix and chain status from LTCFocus.	3,341,487	971(0.03%)

© 2026 Prusynski RA et al. *JAMA Network Open.*

eTable 2. Results of Sensitivity Analysis With SNF Capacity Variables Lagged by One Month

**Associations between lagged skilled nursing facility (SNF) capacity variables within hospital-SNF markets and the hospital length of stay for Medicare fee-for-service beneficiaries discharging from a hospital to a SNF in 2018 and 2019**			
	All Beneficiaries	Dual Eligible Beneficiaries	Non-Dual Eligible Beneficiaries
**Lagged Market SNF Capacity Variables[a]**	**Percent Change in Length of Stay (95% CI)**	**Percent Change in Length of Stay (95% CI)**	**Percent Change in Length of Stay (95% CI)**
SNF Occupancy Rate (10-point increase)	0.5 (-0.8, 1.8)	1.4 (-0.5, 3.3)	0.1 (-1.2, 1.5)
Nursing Staff Hours Per Patient-Day (1-Hour Increase)	-3.9 (-5.8, -1.9)	-3.5 (-6.4, -0.4)	-4.0 (-6.1, -1.7)
Therapy Staffing Hours Per Patient-Day (15-Minute Increase)	0.5 (-1.0, 2.0)	0.9 (-1.1, 2.8)	0.2 (-1.3, 1.8)

[a] SNF capacity variables are created from the month prior to hospital admission, weighted by the proportion of beds each SNF contributes to the market, and then averaged across all SNFs in each discharging hospital's market. Linear regression models were adjusted for all patient and hospital stay characteristics in Table 2 as well as study month and hospital-SNF market fixed effects. Standard errors account for clustering at the hospital and network levels.

© 2026 Prusynski RA et al. *JAMA Network Open.*
