# Supplementary material for: Skilled Nursing Facility Network Capacity and Hospital Length of Stay
Source: JAMA Netw Open. 2026 Apr 30;9(4):e269930. doi: 10.1001/jamanetworkopen.2026.9930 (PMC13133692; doi:10.1001/jamanetworkopen.2026.9930)
Supplement: Supplement 2. — Data Sharing Statement [file jamanetwopen-e269930-s002.pdf]

Data Sharing Statement

Prusynski. Skilled Nursing Facility Network Capacity and Hospital Length of Stay. *JAMA Netw Open*. Published April 30, 2026. doi:10.1001/jamanetworkopen.2026.9930

Data
Data available: No

Additional Information
Explanation for why data not available: Data for this study may not be shared because they are subject to a data use agreement with the Centers for Medicare & Medicaid Services which prohibits the sharing of research identifiable files. Other researchers may access the data used for this study through individual data use agreements with CMS.
